# The Strategy of Predator Evasion in Response to a Visual Looming Stimulus in Zebrafish (*Danio rerio*)

**DOI:** 10.1093/iob/obaa023

**Published:** 2020-08-10

**Authors:** A McKee, M J McHenry

**Affiliations:** Department of Ecology and Evolutionary Biology, University of California, 321 Steinhaus Hall, Irvine, CA 92697, Irvine

## Abstract

A diversity of animals survive encounters with predators by escaping from a looming visual stimulus. Despite the importance of this behavior, it is generally unclear how visual cues facilitate a prey’s survival from predation. Therefore, the aim of this study was to understand how the visual angle subtended on the eye of the prey by the predator affects the distance of adult zebrafish (*Danio rerio*) from predators. We performed experiments to measure the threshold visual angle and mathematically modeled the kinematics of predator and prey. We analyzed the responses to the artificial stimulus with a novel approach that calculated relationships between hypothetical values for a threshold-stimulus angle and the latency between stimulus and response. These relationships were verified against the kinematic responses of zebrafish to a live fish predator (*Herichthys cyanoguttatus*). The predictions of our model suggest that the measured threshold visual angle facilitates escape when the predator’s approach is slower than approximately twice the prey’s escape speed. These results demonstrate the capacity and limits to how the visual angle provides a prey with the means to escape a predator.

## Abstract (es)

Una diversidad de animales sobrevive a los encuentros con los depredadores al escapar de un inminente estímulo visual. A pesar de la importancia de este comportamiento, generalmente no está claro cómo las señales visuales facilitan la supervivencia de una presa de la depredación. Por lo tanto, el objetivo del presente estudio fue comprender cómo el ángulo visual que el depredador subtiende en el ojo de la presa afecta la distancia del pez cebra adulto (*Danio rerio*) de los depredadores. Realizamos experimentos para medir el ángulo visual del umbral y modelamos matemáticamente la cinemática del depredador y la presa. Analizamos las respuestas al estímulo artificial con un enfoque novedoso que calculaba las relaciones entre los valores hipotéticos para un ángulo umbral-estímulo y la latencia entre el estímulo y la respuesta. Estas relaciones se verificaron contra las respuestas cinemáticas del pez cebra a un depredador de peces vivos (*Herichthys cyanoguttatus*). Las predicciones de nuestro modelo sugieren que el ángulo visual del umbral medido facilita el escape cuando el enfoque del depredador es más lento que aproximadamente el doble de la velocidad de escape de la presa. Estos resultados demuestran la capacidad y los límites de cómo el ángulo visual proporciona a una presa los medios para escapar de un depredador.

## Abstract (de)

Diverse Tierarten überleben Begegnungen mit Raubtieren, indem sie einem ansichankommenden visuellen Form entkommen. Trotz der Bedeutung dieses Verhaltens ist es im Allgemeinen unklar, wie visuelle Signale das Überleben einer Beute vor Raubtieren beeinflussen. Ein Ziel der vorliegenden Studie war daher zu verstehen, wie der vom Raubtier gerichtete Blickwinkle auf das Auge der Beute die Entfernung der Beute (ein erwachsenen Zebrafisches (*Danio rerio*) von den Raubtieren beeinflusst. Wir haben Experimente durchgeführt, um den Schwellenwinkel zu messen, und die Kinematik von Raubtier und Beute mathematisch zu modellieren. Wir analysierten die Reaktionen auf den künstlichen Stimulus mit einem neuartigen Ansatz, der die Verhaeltness zwischen hypothetischen Werten für einen Schwellen-Stimulus-Winkel und der Latenz zwischen Stimulus und Reaktion berechnete. Diese Beziehungen wurden gegen die kinematischen Reaktionen von Zebrafischen auf ein lebendes Fischräuber (*Herichthys cyanoguttatus*) verifiziert. Die Ergebnisse unseres Modells deuten darauf hin, dass der gemessene visuelle Schwellenwinkel das Entkommen erleichtert, wenn der Anflug des Raubtiers langsamer ist als ungefähr die doppelte Fluchtgeschwindigkeit der Beute. Diese Ergebnisse zeigen die Fähigkeit aber auch die Grenzen, inwiefern der Sichtwinkel einer Beute die Möglichkeit bietet, einem Raubtier zu entkommen.

## Introduction

Evasive prey survive an encounter with a predator when they successfully execute an escape in response to a threatening sensory cue. Throughout this interaction, the distance between predator and prey affects both sensory information and the prey’s prospects for survival (Easter and Nicola 1996; Wainwright et al. 2001; Tammero and Dickinson 2002; Stewart et al. 2013; Cooper and Blumstein 2015; Pita et al. 2015). Fishes respond to a looming visual stimulus with a “fast-start” escape response (Dill 1971; Preuss et al. 2006; Temizer et al. 2015; Dunn et al. 2016) with a direction determined by the relative size and timing of muscle contractions on either side of the body (Foreman and Eaton 1993). It is unclear how visual cues facilitate this escape at sufficient proximity for prey survival. Therefore, the aim of this study was to measure the visual cues that stimulate an escape response and to examine their effect on the distance from predators in zebrafish (*Danio rerio*, Hamilton 1922). 

**Fig. 1:**
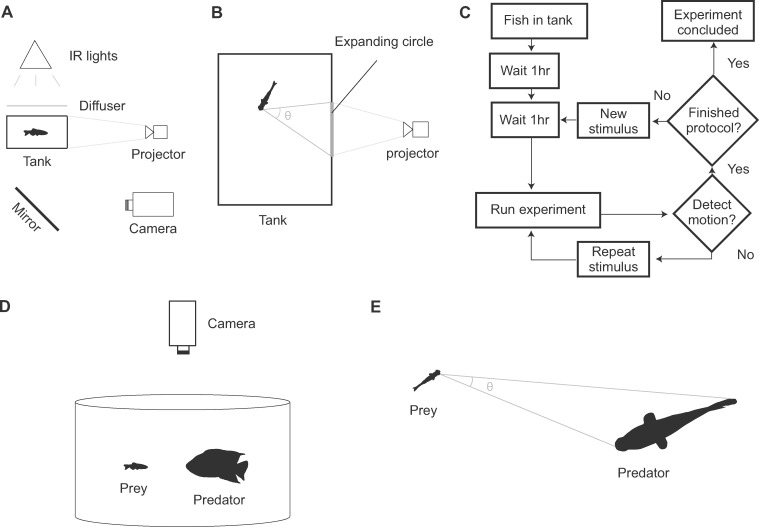
Experimental methods using an artificial and live looming stimulus. (**A**) In the artificial stimulus setup, a camera viewed the underside of the fish in the tank using a mirror at a 45°  angle. The stimulus, a circle of expanding diameter, was projected on the side of the tank by a projector. (**B**) As seen from below, the stimulus presented a visual angle (θ). (**C**) Flow chart for the sequence of automated experiments using the artificial stimulus. Computers controlled the timing of the experiments. (**D**) In experiments with a live predator, an individual zebrafish was introduced into a large circular tank with a red Texas cichlid. (**E**) The visual angle was measured from the eye of the prey to the margins of the predator’s body.

The effects of a threatening visual stimulus can be studied under controlled experimental conditions. Looming may be simulated by projecting an expanding circle upon the wall, floor, or ceiling of a holding tank. In response, prey will generally initiate an escape if the circle’s expanse is sufficiently rapid and large. A number of behavioral studies have considered the particular cue that triggers an escape by recording its timing relative to the projected stimulus in a diversity of animals that includes crabs (Oliva and Tomsic 2012), insects (Santer et al. 2008; Ache et al. 2019) primates (Schiff et al. 1962; Clery et al. 2017), and birds (Wang and Frost 1992). Contemporary studies on fishes suggest that a threshold value of the visual angle offers the most robust predictor of an escape, within a range of approach velocities (Preuss et al. 2006; Temizer et al. 2015; Dunn et al. 2016; Bhattacharyya et al. 2017). The visual angle is the angle subtended on the eye by each of the lateral margins of the looming stimulus (Fig. 1B). The fast-start is characterized by the body rapidly curling into a “C” shape and then unfurling to accelerate (Weihs 1972). In piscivorous interactions, this escape is commonly faster than the speed of the approaching predator, which often brake as they approach the prey, perhaps as a measure to coordinate a suction-feeding strike (Higham et al. 2005; Higham 2007; Stewart et al. 2013, 2014). As a consequence, the minimum distance between predator and prey is often achieved shortly after escape initiation. In this context, “minimum distance” refers to smallest distance attained over time.

Resolving the threshold-stimulus angle poses a challenge for experimentalists because it is generally unknown how much time transpires between the threshold-stimulus and its response. This latency is due to the neurophysiological integration of the visual stimulus and the formulation of a motor response. In the absence of physiological measurements, this latency has been assumed by experimentalists to be either negligible or it has been approximated as a fixed parameter. Differences in this approximation have the potential to yield contrasting results for the threshold-stimulus angle. This is largely due to the visual angle increasing at a nonlinear rate when a predator approaches at a constant velocity. Because of this nonlinearity, small differences in the estimated latency can suggest very different values for the threshold. Indeed, the literature offers a variety of values of the threshold-stimulus angle based on different latency values (Dill 1974; Preuss et al. 2006; Temizer et al. 2015; Dunn et al. 2016).

The threshold-stimulus angle for an escape response has a direct influence on the evasive strategy of a prey fish. Evasion strategies are thought to be either unpredictable or optimized in some manner (Isaacs 1965; Weihs and Webb 1984; Domenici et al. 2011; Combes et al. 2012; Nair et al. 2017). The leading ideas for optimal evasion strategy are based on a differential game theory known as the homicidal chauffeur (Isaacs 1965), which has been applied to fish predator–prey interactions (Weihs and Webb 1984; Soto et al. 2015). This theory includes calculations of the minimum distance (with respect to time) of a prey from a predator approaching from a fixed direction at a constant speed. Prey seek to keep this minimum distance as large as possible with an optimal strategy. The minimum distance depends principally on the relative speed of the predator and prey and the distance between them at the start of the escape. This escape distance is dictated by the threshold-stimulus angle.

This study measured the threshold-stimulus angle in zebrafish and considered its effect on the minimum distance. We hypothesized that zebrafish escape at a threshold that permits sufficient distance from a predator to evade capture. We developed an approach that is novel in a few respects to address some of the technical challenges for a study of this kind. We obtained a sufficient number of experiments without animal habituation with a computer-automated setup that altered its protocol according to the behavioral responses of the animal. The second challenge was to find values for both the threshold visual-stimulus angle and latency that are predictive of the behavior (also for the rate of change of the visual angle, see Supplementary Materials). We developed an analytical approach that uses the statistical power of all of our experiments to resolve a relationship between the threshold-stimulus angle and the latency. We additionally performed experiments on a fish predator, a red Texas cichlid (*Herichthys cyanoguttatus*), to validate the values for the threshold-stimulus angle. Finally, we applied a game-theoretical framework to consider the strategic implications of this threshold.

## Methods

### Animal husbandry

We raised wild-type (AB line) zebrafish (*D. rerio*) according to standard procedures (Westerfield 1993). The fish were held in a flow-through tank system (Aquatic Habitats, Apopka, FL, USA) in 3 L containers at 27°C and fed daily with a 14:10 h light:dark cycle. The cichlid (*H. cyanoguttatus*, 15 cm total length) that we used as a predator was obtained from a fish store and was held separately from the zebrafish at 25°C on the same light cycle and fed daily. All rearing and experimental protocols were conducted with the approval of the Institutional Animal Care and Use Committee at the University of California, Irvine (Protocol #AUP-17-012).

### Responses to a projected looming stimulus

We recorded the behavioral responses of 56 zebrafish exposed to a projected looming stimulus. Individual fish were placed in a rectangular clear acrylic tank (7.5 cm × 18.5 cm floor, water depth of 7 cm). The walls were angled outward by 4°  to minimize their appearance when viewed from below. The tank was elevated above a mirror tilted at a 45°  angle from the view of a high-speed camera (Photron FastCam SA2, San Diego, CA, USA set to 1000 fps at 1280 × 640 pixels) with a 55 mm macro lens (Nikon Corporation, Melville, NY, USA). The lens was positioned at a distance from the closest tank wall (54 cm) that allowed us to view the entire underside of the tank through the mirror. Three infrared lights (IR Illuminator CM-IR200, CMVision, Houston, TX, USA; wavelength: 850 nm, illuminance: 10 lux) were placed above the tank and a plastic lid placed on the tank served as a light diffuser. Another diffuser was affixed to the wall of the tank facing the camera and a small projector (Brookstone 801143 Texas Instruments, Merrimack, NH, USA) was focused on this surface to present the looming stimulus (Fig. 1A).

The experiments were conducted with an automated system operated by two computers to allow for high-throughput experimentation. One computer was used to turn on the IR lights, initiate recording by the video camera, and to project the looming stimulus. These tasks were achieved with custom software scripted in MATLAB (version R21015a, Mathworks, Natick, MA, USA). The IR lights were controlled using an analog output channel from a data acquisition device (DAQ, National Instruments NI DAQ USB-6009, Austin, TX, USA) attached to a solenoid switch controlling the power to the lights. The DAQ also triggered the camera, which was configured by the second computer running the camera software (Photron FASTCAM Viewer 3, San Diego, CA, USA). After an experiment, the second computer also ran custom MATLAB software to perform a kinematic analysis on the video recording to determine whether the fish responded to the stimulus. The two computers shared a network connection, which allowed the kinematic results of an experiment determined by one computer to be communicated to the other computer, which controlled the stimulus. This allowed the result of an experiment to affect the decision about the following experiment. This automated setup offered the additional benefit of eliminating the presence of an experimentalist that could influence the fish’s behavior.

Our automated experiments followed a protocol that aimed to maximize the number of responses we recorded from an individual fish (Fig. 1C). The zebrafish was permitted to acclimate (2 h) prior to the first experiment and then experiments were performed once every hour, which pilot experiments demonstrated was a long-enough interval to prevent habituation (Randlett et al. 2019). Before each experiment, IR illumination was turned on 2 min prior to the presentation of a visual stimulus. The stimulus initially consisted of a small dark circle on a white field that was animated with lateral oscillations for 3 s to attract the attention of the fish. The circle then expanded in diameter until it reached its final size, which completely enveloped the screen. The camera was triggered to begin recording as the circle commenced expansion and to record for a duration of 3.99 s. Our analysis software automatically determined whether the fish responded by tracking the velocity of the center of the body. If the fish responded, a different stimulus was presented in the next experiment. Otherwise, fish were assumed not to have seen the stimulus and the same stimulus was repeated without the 1 h delay to ensure the stimulus was visible to the fish during the recording. This process continued until the fish had been exposed to eight unique stimuli and two controls (no stimulus shown) or until 24 h had transpired.

The rate of change of the diameter of the looming stimulus was varied to simulate a virtual predator approaching at a variety of fixed speeds. This virtual predator was assumed to have a circular appearance of fixed diameter ($S_{\text{vir}}=30\;\text{cm}$) and to move toward the screen at a constant speed ($u_{\text{vir}}$). If one assumes that the observer maintains their distance from the wall ($d_{\text{wall}}=2\;\text{cm}$) upon which the stimulus is projected, then the diameter of the projected circle ($S_{\text{wall}}$) may be calculated through an application of similar triangles:

(1) $$\frac{S_{\text{vir}}}{d_{\text{vir}}}=\frac{S_{\text{wall}}}{d_{\text{wall}}},$$

where $d_{\text{vir}}$ is the distance between the prey and virtual predator, which may be calculated as $d_{\text{vir}}=d_{\text{vir},0}-u_{\text{vir}}t$. The initial distance of the virtual predator ($d_{\text{vir},0}$) was determined with Equation 1 using the value for the diameter of the attractive stimulus ($S_{\text{wall},0}=5\;\text{mm}$). Our series of experiments projected stimuli intended to simulate virtual predators that approach at the following velocities: $19.35{\;\text{cm}\;s}^{-1},\;25.8{\;\text{cm}\;s}^{-1},\;32.25{\;\text{cm}\;s}^{-1},\;38.7{\;\text{cm}\;s}^{-1},\;45.15{\;\text{cm}\;s}^{-1},\;51.6{\;\text{cm}\;s}^{-1},\;58.05{\;\text{cm}\;s}^{-1}$, and $64.5{\;\text{cm}\;s}^{-1}$. Expressed by the ratio of the stimulus radius to its approach velocity, these stimuli ranged from 230 ms to 780 ms. However, the prey fish was free to move in these experiments which violates the assumptions of a fixed distance and position of the viewer. We therefore measured the realized visual angle to which the fish were exposed in each experiment, as detailed below.

### Kinematic analysis

We measured the visual angle (θ) to which the fish were exposed prior to their escape response. This was found from measurements of the fish’s position from our video recordings with a MATLAB program that first found the body of the fish as a dark area of pixels by thresholding each video frame. The fish was differentiated from pixels of similar intensity by its area and the midline of the body was found as a line of pixels furthest from the periphery of the body using distance mapping (the “bwdist” function in MATLAB). The greater width of the body at its anterior end differentiated it from the posterior end of the body. The position of the fish’s eyes was identified by their consistent distance from the rostrum. We calculated the visual angle (θ) presented by the looming stimulus using the center of the eye and the width of the dark circle (Fig. 1B) for all frames in which the circle was within the eye’s field of view (Pita et al. 2015). Owing to the relatively modest overlap in the visual field of the two eyes, the stimulus rarely was presented within view of both eyes. In such instances, we considered only the visual angle of greater magnitude. We examined only those experiments that successfully elicited a fast start where the body of the fish curled into a “C” shape prior to its acceleration and we pooled the measurements from all fish.

**Fig. 2:**
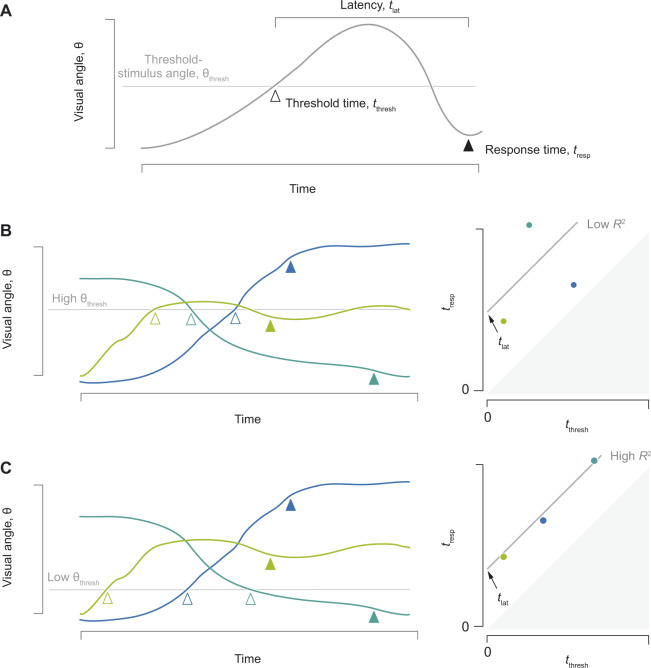
Analytical method for testing values for the threshold-stimulus angle. (**A**) Measurement of the visual angle for a hypothetical experiment with annotations for the response time ($t_{\text{resp}}$, filled triangle) and threshold time ($t_{\text{thresh}}$, open triangle), when the threshold angle was exceeded. The threshold time was estimated by assuming a particular value for the threshold visual angle (θ_thresh_) and the latency ($t_{\text{lat}}$) was determined as the difference between stimulus and response times. (**B** and **C**) The same three experiments (denoted by the colored lines) were analyzed assuming a high (B) and low (C) threshold stimulus, given measured values for the visual angle and response time (denoted by filled triangles, left plots). The first time the visual angle exceeds the threshold-stimulus angle is the threshold time (denoted by empty triangles, left plots). As detailed in the text, the relationship between the stimulus and response times (right plots) should conform to a linear relationship with a slope of unity and a *y*-intercept equal to the latency. In this example, a better fit to this relationship was obtained by assuming a low threshold (C) than a high threshold (B), as would be indicated by the coefficient of determination (*R*^2^, right plots).

We used a novel analytical method to determine the relationship between the threshold-stimulus angle and latency that was most consistent with our experimental results. Each experiment provided measurements of the visual angle and the response time ($t_{\text{resp}}$), the moment when the escape response was initiated. From these measurements, we performed a series of calculations to determine the threshold time ($t_{\text{thresh}}$), the moment when the stimulus reached threshold, and the value of the threshold-stimulus angle (θ_thresh_). We considered a range of hypothetical values for the threshold-stimulus angle and for each value, the threshold time was calculated as the moment at which the measurements of visual angle exceeded the threshold (Fig. 2A). Such calculations were performed to yield a relationship between the values of threshold time and response time for all experiments (Fig. 2B). If the latency between stimulus and response ($t_{\text{lat}}$) is consistent among experiments, then one should predict this relationship to be linear, with a slope of unity (i.e., $t_{\text{resp}}=t_{\text{thresh}}+t_{\text{lat}}$). For each hypothetical value of the threshold-stimulus angle, we calculated values for the threshold time among all experiments and performed a least-squares linear fit for the intercept for an assumed a slope of unity. We used the coefficient of determination (*R*^2^) as a metric of the fit of this relationship to the data. This was achieved for 100 values at equal intervals for the visual angle (0.5° ≤ θ_thresh_ ≤ 25.0°). The product of this process was a relationship between the threshold-stimulus angle and latency that matched our experimental results and an indication of which threshold values offered the best fit for the data. This general approach may be applied to other sensory cues, such as the rate of change in the visual angle (see Supplementary Materials).

### Responses to a live predator

We compared the responses to a projected stimulus to those elicited by a live cichlid predator (*H. cyanoguttatus*). We positioned a high-speed video camera above a cylindrical tank (ø=90 cm, 760 L, Fig. 1D), which was surrounded with a tarp to conceal the experimentalist. The cichlid was placed in the tank and allowed to acclimate for 2 h before we introduced a zebrafish to the tank. The camera recorded continuously on a loop until manually-triggered to save at the end of an escape response from the zebrafish. This escape generally occurred after several minutes of the zebrafish’s introduction. Two escapes were recorded from each of 48 zebrafish and the cichlid never succeeded in capturing these prey.

The videos were manually digitized with ImageJ (version 1.52a, Wayne Rasband, National Institutes of Health, USA) to measure the coordinates of the rostrum and tail of both the cichlid predator and the zebrafish. From these coordinates, we calculated the visual angle between the anterior end of the zebrafish and the span of the cichlid’s body (Fig. 1E). These coordinates were obtained for each frame from at least 150 ms prior to when the zebrafish initiated a C-start escape until the zebrafish either was coasting or swimming in its ultimate direction (∼50 ms after the end of Stage 2).

We used the results of these experiments as an indication of how the responses to the projected stimulus applied to encounters with a live predator. For each experiment with the cichlid, we considered the values for the visual angle prior to an escape as the possible values that triggered the response. For each of these values, we calculated the time between the time of the possible trigger and the escape time and used this as our proposed latency. This generated a range of hypothetical values for both the threshold and latency among all experiments. We compared these values against the latency and threshold values obtained in response to the projected stimulus. We considered overlapping values from the two types of experiments as indication of the responses that best apply to a live predator for zebrafish.

### Mathematical modeling

We used a mathematical model to evaluate how the threshold-stimulus angle affects the minimum distance between predator and prey. This allowed for a consideration of strategy beyond the kinematics of the predator species considered presently. Our analysis was particularly concerned with the minimum distance attained by the predator because that value represents the best opportunity for prey capture. This agent-based model calculated the kinematics of predator and prey with simplified motion. The prey was assumed to be motionless until stimulated to initiate an escape and the predator moved at a constant velocity that was directed toward the initial position of the prey. The first step in such calculations required finding the distance between predator and prey. The visual angle of a predator of width *w* was calculated from the prey’s perspective on the approach with the following relationship:

(2) $$\text{tan}\;\left(\frac{\theta }{2}\right)=\frac{w}{2d}.$$

Solving Equation 2 where $d=d_{\text{thresh}}$ allowed us to calculate the threshold distance as a function of the threshold visual angle ($\theta =\theta_{\text{thresh}}$). The response distance at the time of the escape was found by considering the reduction due to the prey’s latency ($d_{\text{resp}}=d_{\text{thresh}}-ut_{\text{lat}}$, where *u* is the predator’s velocity). The maximum possible value for the threshold visual angle was obtained from Equation 2 with distance set to the minimum response distance, equal to the product of predator speed and prey latency:

(3) $${\left(\theta_{\text{thresh}}\right)}_{\text{max}}=2\arctan\left(\frac{w}{2ut_{\text{lat}}}\right).$$

The minimum distance depends on the kinematics of a prey’s escape response. The fast-start allows a fish to attain rapid speeds in a brief period of time. In a preliminary analysis, we varied speed over time, but found that our predictions were similar to an instantaneous onset of the mean escape speed measured in response to the live predator ($v=3.7\;{\text{cm}\;s}^{-1}$, *N *=* *63). The fast-start is capable of sending the fish in different directions with respect to the predator, expressed by the escape angle (α). When the predator is slower than the escaping prey, the minimum distance will equal the response distance for low escape angles. These values for minimum distance are expressed by the following equation (Soto et al. 2015):

(4) $$d_{\text{min}}=d_{\text{resp}}\;\text{if}\;|\alpha |\leq \arccos(K),$$

where *K* is the ratio of predator to prey speed (K=u/v). However, if the predator was faster than the prey, or the escape angle was greater than arccos(*K*), we calculated the minimum distance using a previously-developed formulation (Weihs and Webb 1984; Soto et al. 2015) that assumes fixed velocities for the predator and prey:

(5) $$d_{\text{min}}=\sqrt{\frac{d_{\text{resp}}^{2}\;\text{sin}\;{\left(\alpha \right)}^{2}}{K^{2}-2K\;\text{cos}(\alpha )+1}}.$$

Using Equations 4 and 5, we calculated the minimum distance for when a predator is slower (*K *=* *0.5), slightly faster (*K *=* *1.5), and much faster (*K *=* *3.0) than the prey. At each speed, we examined the effects of six values of the escape angle (0≤α≤160 deg). In separate calculations, we varied predator speed (0<K<5) to simulate a visual angle stimulus (θ_thresh_ = 14°). Throughout, small values for the minimum distance ($d_{\text{min}}<2\;\text{cm}$) were considered threatening to the prey, based on previous work on the suction feeding of predatory cichlids that are comparable in size to our cichlid predator (Wainwright et al. 2001).

## Results

**Fig. 3:**
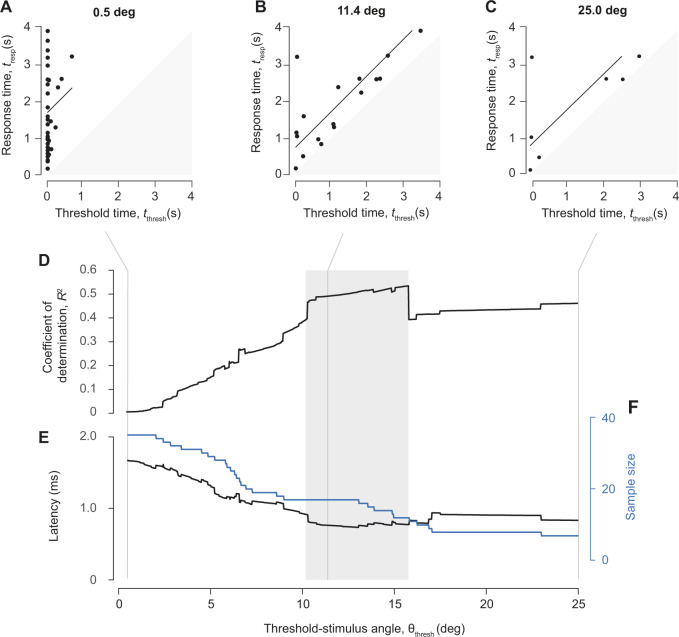
Determination of the threshold visual angle for all experimental responses to a projected looming stimulus. (**A**–**C**) Relationship between the threshold and response times for three representative values for the threshold visual angle. As described above (Fig. 2), this relationship is predicted to conform to a slope of unity, with a *y*-intercept equal to the latency predicted for each value of the threshold-stimulus angle. (**D**) The coefficient of determination for the unity-line fit for each value of the threshold-stimulus angle, (**E**) the corresponding latency, and (**F**) sample size (blue) are plotted. Experiments with an escape occurring before the threshold-visual angle was seen were removed, resulting in a decreasing sample size as threshold-visual angle increases. We selected values for latency and the threshold visual angle where the coefficient of determination was relatively high (gray bar) for comparison with responses to a live predator (Fig. 4).

We evaluated the behavioral responses to an artificial stimulus with a novel analytical method. As detailed above, we measured the visual angle prior to an escape response in each experiment. By assuming the latency between the stimulus and response among experiments, we found the response time as the sum of the threshold time and the latency (Fig. 2). We examined how our measurements compared to this relationship at variable threshold values for the visual angle (Fig. 3A). This analysis considered only experiments where the hypothetical threshold stimulus was attained within a recording prior to the response. Because of this, each value of θ_thresh_ is reported with its corresponding sample size (Fig. 3F). As reflected by the coefficient of determination, we found the best matches for a range of threshold values for the visual stimulus (10.3° < θ_thresh_ < 15.8°, 12<N<17, Fig. 3D). These threshold values correspond to a range in latency between 740 ms and 780 ms (Fig. 3E).

**Fig. 4:**
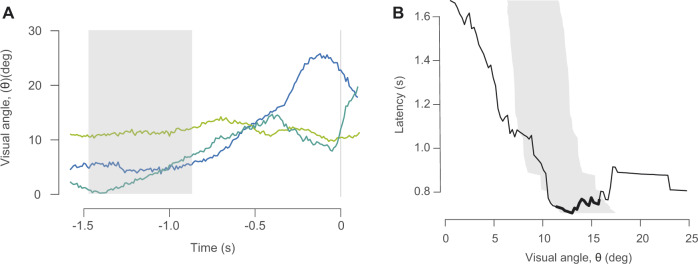
Responses to a live predator. (**A**) Three representative measurements of the visual angle (colored lines), with time calculated relative to the response time (gray line). We considered all values for the range of latency values (gray bars) suggested by measured responses to a projected stimulus (Fig. 3). (**B**) Comparison of measurements from the projected-stimulus experiments (black curves) and live-predator experiments (gray areas) for the threshold-stimulus angle. The margins for the live predator are the first and third quartiles of values for the threshold-stimulus angle at each value for the latency. The regions of the threshold-stimulus angle with a high coefficient of determination (gray bars in Fig. 3) which fall within the bounds of the live-predator experiments are highlighted (heavy black curves).

We considered the escape responses of zebrafish to a live predator. Our measurements for the visual angle prior to an escape varied largely due to the relatively rapid movements of the zebrafish (Fig. 4A and B). These measurements represent hypothetical threshold cues that stimulated an escape response to the live predator, which we examined for the range of latency values recorded for the projected stimulus (Fig. 3E). For each value of the latency, we calculated the first and third quartiles for all measurements of the visual angle. The first quartile was as low as 6.3°  (*N *=* *63) and the third quartile did not exceed 17.4° across values of latency (Fig. 4C). This quartile range encompassed most of the values measured in response to the projected stimulus that showed a high coefficient of determination (11.29° < θ_thresh_ < 15.8°).

**Fig. 5:**
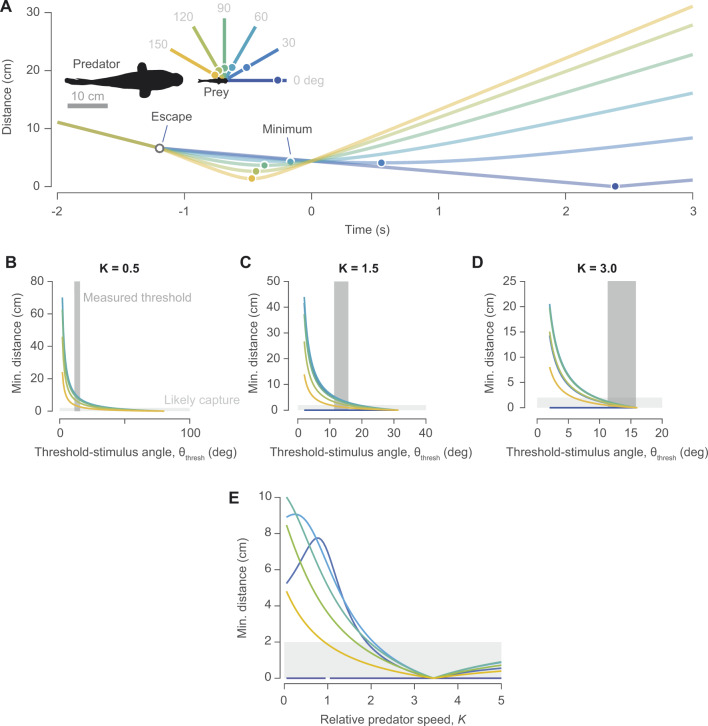
The effects of the threshold-stimulus angle on the evasion strategy of zebrafish. (**A**) Numerical simulations of kinematics of predator and prey show the distance between the predator’s rostrum and prey’s center of body over time for escape directions between 0° and 150° where relative predator speed (*K*) =1.5, the latency time ($t_{\text{lat}}$) =0.8 s, the threshold angle (θ_thresh_) = 10°, predator size (*S*) =2.5 cm, and prey velocity (*V*) =3.7 cm s^–1^. Time = 0 s is when collision between predator and prey would have occurred if escape had not been initiated. The inset shows the relative position of predator and prey at the start of the escape and the radiating lines show the prey’s trajectory for differing escape direction with the same color coding as all other panels. The minimum distance occurs at the position of the filled circles in both the graph and insert. (**B**–**D**) The minimum distance predicted (Equation 5) for varying threshold values for the visual angle. The vertical bars (dark gray) indicate the range of threshold values favored by our analysis of experiments (Fig. 4). The horizontal bars (light gray) indicate distance values where the prey have a low probability of escape ($d_{\text{min}}<2$ cm). Calculations were performed for predators of variable relative speed (*K *=* *0.5, *K *=* *1.5, and *K *=* *3.0). (E) The minimum distance as a function of relative predator speed at particular values of the threshold visual angle (θ_thresh_ = 14°).

We used a mathematical model to examine the strategic implications of our measured responses to a looming visual stimulus. As detailed above, our model considered the distance between predator and prey, with particular focus on the minimum distance as the best opportunity for prey capture. In this analysis, minimum distance values of <2 cm were considered to offer a high probability of capture in accordance with prior work (Wainwright et al. 2001). This model assumes that the prey remains motionless until initiating an escape, at which point they escape at a fixed velocity (Fig. 5A). By also assuming a fixed velocity for the predator, we were able to calculate the minimum distance predicted for a range of threshold values, escape angles, and relative speed of the predator (Equation 5). In all cases, the minimum distance was predicted to decrease asymptotically toward zero with increases in the threshold-stimulus angle. As a consequence of this non-linear relationship, small differences at the low-end of threshold values were found to have relatively large effects on the minimum distance. As a result, minimum distance values greatly exceeded the proximity at which a suction-feeding predator may typically strike (Fig. 5B–D). Within the range of threshold-stimulus angle that we measured, the minimum distance exceeded 2 cm for all but the smallest escape angles if the predator was slower (Fig. 5B) or just slightly faster (Fig. 5C) than the prey. Our calculations suggest that prey will likely fail to escape the predator at all escape angles if the predator’s approach is more than twice as fast as the escaping prey for a predator of width comparable to the cichlid (*w *=* *2.5 cm, Fig. 5E). Similar results were obtained for a predator that was twice as wide (Supplementary Fig. S4G), but a substantially more narrow predator would likely succeed in capturing prey at almost all speeds (Supplementary Fig. S4H).

## Discussion

Our results demonstrate the strategic implications of responses to a looming stimulus by zebrafish. Using a novel analytical method, we found relationships between latency and the threshold-stimulus angle (Fig. 3) to an artificial stimulus. We related these results to a live predator (Fig. 4) and considered their significance to strategy via mathematical modeling (Fig. 5). Our results suggest that zebrafish have a strategic advantage when they respond to the measured threshold-stimulus angle. However, the effectiveness of this response is reduced for relatively fast predators. Our findings offer a strategic basis for understanding the neurophysiology of visual processing and motor commands for the escape responses of fishes.

Our experimental approach addresses a challenge to inferring a sensory cue from behavioral experiments. This challenge emerges from the unknown latency between stimulus and response, which creates ambiguity in the magnitude of the stimulus intensity at the moment the response was stimulated. This latency, which is due to the neurophysiological integration of the visual stimulus and formulation of a motor response, is easily resolved in experiments that consider a discrete stimulus, such as a step-change in light intensity, and an escape response (Lin and Nash 1996; Burgess and Granato 2007). In contrast, the latency is less clear for a stimulus like the looming appearance of a predator, where the visual angle increases over time. Due to non-linearity in the visual angle of a looming stimulus, small differences in an estimate for latency may yield contrasting values for the threshold-stimulus angle. Our approach determines when a particular threshold-stimulus angle was reached, given the timing of the response and the measurements of the visual angle (Fig. 2A). By assuming a consistent latency among all experiments, the response time was presumed equal to the sum of the threshold time and latency. We evaluated how well measurements of response and threshold-stimulus times conformed to this relationship using the coefficient of determination (Fig. 2B and C). By that standard, we found that about half of the variation in response time could be predicted from measurements of the visual angle (Fig. 3D). This method yielded a set of values for hypothetical sensory cues and their corresponding latency values, which contrasts the conventional practice of assuming a solitary value for the latency (Gabbiani et al. 1999; Oliva and Tomsic 2012; Temizer et al. 2015; Ache et al. 2019).

The present results may be compared to previous studies of similar experimental design. Adult goldfish were found to respond principally to a visual angle of ∼21° ($t_{\text{lat}}=35$ ms) (Preuss et al. 2006), which is roughly half as sensitive as what we observed (Fig. 3). The goldfish results are consistent with findings from one study on zebrafish larvae ((θ_thresh_ = 21.7°, $t_{\text{lat}}=35$ ms) (Temizer et al. 2015). However, another study on larvae found sensitivities that were less than a third of these values (θ_thresh_ ∼ 72°, $t_{\text{lat}}=81$ ms) (Dunn et al. 2016). Differences in methodology, such as whether the fish were permitted to swim freely and the assumed value for the latency, may account for these disparate results. We found a lower threshold for the visual angle in adults (Fig. 3) than the studies on larvae, which could be related to differences in predator types between the two groups. A recent study has shown that contrast in addition to visual size is an important parameter that fish use to determine when to escape from a looming stimulus (Coronel et al. 2020).

We considered the implications of the threshold-stimulus angle on the evasion strategy of zebrafish through an application of differential game theory (Weihs and Webb 1984; Soto et al. 2015). Our model calculated the minimum distance attained between the predator and prey, assuming a fixed velocity for both fish (Fig. 5A). Reductions in the visual angle (Fig. 5B–D) show disproportionate increases in the minimum distance, due to their nonlinear relationship. Our experiments suggest that zebrafish respond to visual angles where they are predicted to successfully evade predators, provided the predator is relatively slow (Fig. 5B and C). However, the prospects for survival declined precipitously if the predator approached the prey at more than twice the escape speed (Fig. 5E). This supports our hypothesis that zebrafish escape from a threshold visual angle that allows them to remain at a safe distance from an approaching predator. By varying the size of the predator, we found that narrower predators had an advantage over wider predators (Supplementary Fig. S4), because the narrower predator can approach a closer distance to the prey before the threshold-visual angle is detected by the prey.

The result of a prey’s evasion strategy depends on the actions of the predator. Although predator fish are generally capable of faster swimming than prey, it is common for suction-feeding predators, such as the cichlid considered presently, to approach their prey slowly. Many species actively brake on the approach by expanding their pectoral fins (Higham et al. 2005; Higham 2007). Suction feeding offers a brief and spatially-limited opportunity to capture prey (Day et al. 2005; Holzman et al. 2007) and it could be that braking enhances the precision of a strike. By contrast, the fast-start is the most rapid swimming of which a fish is capable and may therefore routinely exceed the speed of a suction-feeding predator (Stewart et al. 2013, 2014). Therefore, slow predators may be common in many predator–prey encounters. Relying on the visual angle to stimulate an escape may therefore be successful for prey like zebrafish when they encounter a variety of suction-feeding fish predators.

In summary, we found responses to the visual angle from behavioral responses of zebrafish adults to a projected looming stimulus and live predator. By modeling the kinematics of predator and prey, we considered how these responses affect the evasion strategy of zebrafish. These calculations illustrate how the visual angle provides a robust sensory cue for escaping predators at sufficient distance for a high probability of survival. However, our results also demonstrate the limits to the measured threshold-stimulus angle, which generally fails when the predator is more than twice as fast as the prey (Fig. 5E). This combination of experimentation and mathematical modeling has the potential to reveal how sensory cues affect the strategy of both predator and prey in a diversity of animals.

## Supplementary Material

obaa023_Supplementary_Data
