# Supplementary material for: The Strategy of Predator Evasion in Response to a Visual Looming Stimulus in Zebrafish (Danio rerio)
Source: Integr Org Biol. 2020 Aug 10;2(1):obaa023. doi: 10.1093/iob/obaa023 (PMC7750966; doi:10.1093/iob/obaa023)
Supplement: obaa023_Supplementary_Data [file obaa023_supplementary_data.pdf]

Supplemental Materials

We analyzed the responses of prey to the rate of change of the visual angle (θ') of a looming stimulus. The consensus of the contemporary literature favors the visual angle as more predictive of an escape than its rate (Temizer et al., 2015; Dunn et al., 2016; Preuss et al., 2006; Bhattacharyya et al., 2017). Nonetheless, the rate of change was previously considered the primary cue to initiate a startle response (Dill, 1974) and may provide a high-order influence on the timing of an escape (Dunn et al., 2016; Bhattacharyya et al., 2017). We additionally performed this analysis to demonstrate the ability of our analytical approach to consider sensory cues other than the visual angle.

In the analysis outlined in Methods, we considered the maximum possible value for a sensory cue to bound the theoretical possibilities. To find the maximum value for the threshold rate of change for the visual-angle, we solved Eqn. 2 where $t_{\text{thresh}} = -t_{\text{lat}}$ to yield the following relationship:

$$\left(\theta'_{\text{thresh}}\right)_{\max} = \frac{4wu}{w^2 + 4t_{\text{lat}}^2 u^2}. \tag{S1}$$

Finding the escape distance for a threshold value of the visual-angle rate required consideration of the timing of the escape. For this, we solved Eqn. 2 for the visual angle as a function of time ($\theta(t)$), zero equal to the time-to-collision, and negative time values on the approach. Using this equation, we found the first-derivative of the visual angle with respect to time to calculate the time at which the threshold-stimulus angle rate was reached:

$$t_{\text{thresh}} = -\frac{\sqrt{w(4u - w\theta'_{\text{thresh}})}}{2u\sqrt{\theta'_{\text{thresh}}}}. \tag{S2}$$

The distance between the predator and prey was calculated ($d_{\text{resp}} = -u(t_{\text{thresh}} + t_{\text{lat}})$) for the moment at which the prey initiated their escape at varying threshold values.

As performed for the visual angle (Fig. 3), we determined the threshold values for the latency and visual-angle rate of change in response to an artificial stimulus (Fig. S1). We calculated the visual-angle rate of change discretely from measurements of the visual angle that were smoothed with a spline (the 'spaps' function in MATLAB). This entailed finding the best fit to the unity line over a range of values (15.3 deg s^{-1} $< \theta'_{\text{thresh}} <$ 27.5 deg s^{-1}, $17 < N < 26$) that corresponded to the average of values for latency (830 ms $<$ $t_{\text{lat}} <$ 850 ms) that were about one-tenth of a second longer than obtained for the visual angle.

The behavioral responses to the artificial stimulus was tested against experiments with a live predator. The results for the threshold visual-angle rate of change found variation

between quartiles of -481.6 deg s^{-1} and 511.1 deg s^{-1} in response to the live predator (Fig. S2B). This wide range of variation included all the values measured in response to the projected stimulus with a high coefficient of determination (15.3 deg s^{-1} $< \theta'_{\text{thresh}} <$ 27.5 deg s^{-1}). This indicates general agreement between the results of the two types of experiments and is consistent with previous estimates of 24.6 deg s^{-1} ($t_{\text{lat}} = 0$ ms) (Dill, 1974).

We modeled the kinematics of predator and prey where the prey responds to a threshold value of the rate of change in the visual angle. Simulation results suggested that zebrafish escaped at a minimum distance that shows a low likelihood of escape, regardless of the predator's speed (Fig. S3). This suggests that the visual-angle rate of change does not offer a robust sensory cue for successful evasion, which is unlike what is predicted for the visual angle (Fig. 5).

References

Bhattacharyya, K., McLean, D. L. and MacIver, M. A. (2017). Visual threat assessment and reticulospinal encoding of calibrated responses in larval zebrafish. *Current Biology* **27**, 2751–2762.

Dill, L. M. (1974). The escape response of the zebra danio (brachydanio rerio) i. the stimulus for escape. *Animal Behavior* **22**, 711–722.

Dunn, T. W., Gebhardt, C., Naumann, E. A., Riegler, C., Ahrens, M. B., Engert, F. and Del Bene, F. (2016). Neural circuits underlying visually evoked escapes in larval zebrafish. *Neuron* **89**, 613–628.

Preuss, T., Osei-Bonsu, P. E., Weiss, S. A., Wang, C. and Faber, D. (2006). Neural representation of object approach in a decision-making motor circuit. *Journal of Neuroscience* **26**, 3454–3464.

Temizer, I., Donovan, J. C., Baier, H. and Semmelhack, J. L. (2015). A visual pathway for looming evoked escape in larval zebrafish. *Current Biology* **25**, 1823–1834.

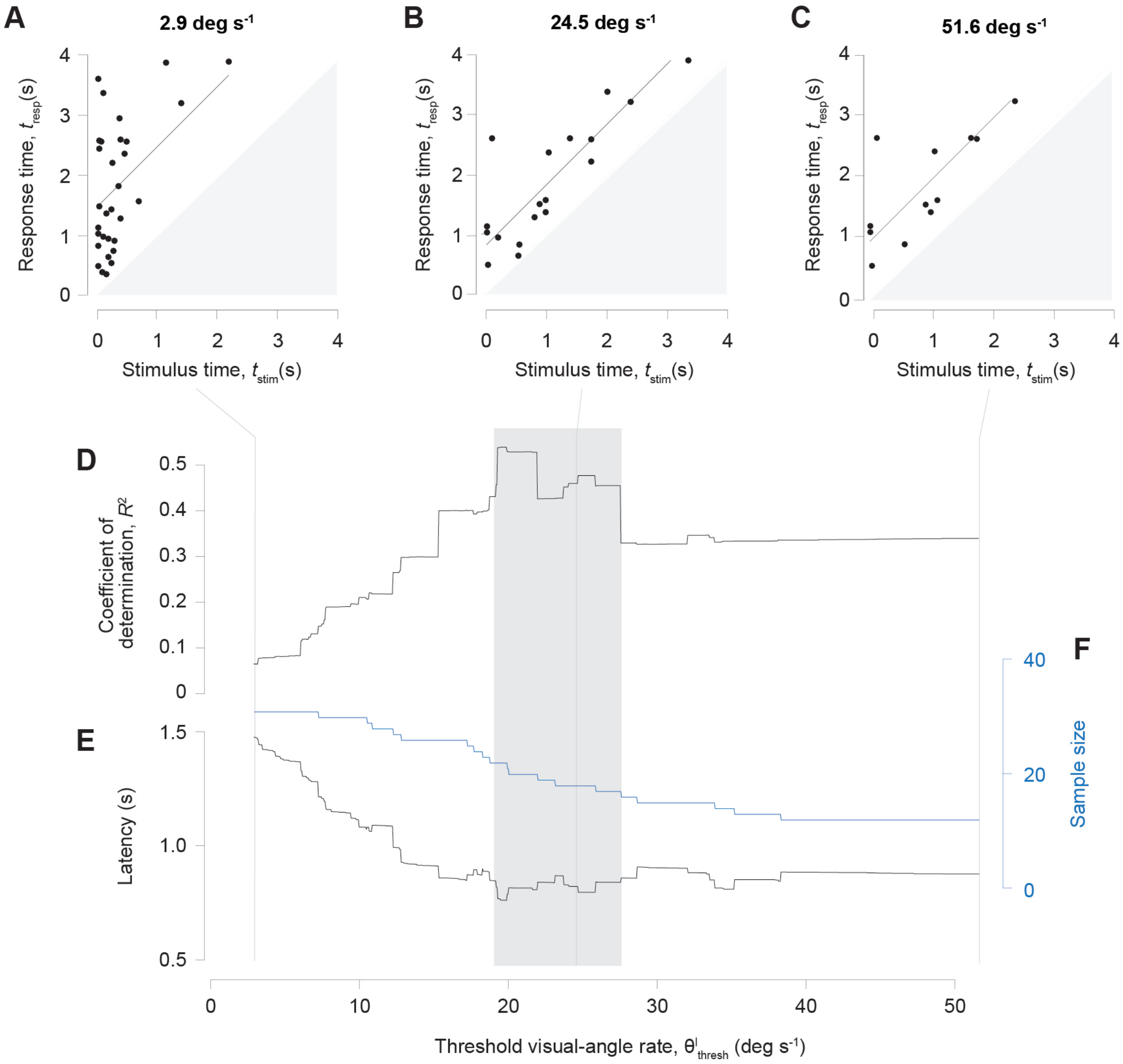

Figure S1: Determination of the threshold visual-angle rate of change for experimental responses to the projected looming stimulus. (A–C) Relationship between the stimulus and response times, assuming three different values for the threshold visual angle rate. As described in the present manuscript (Fig. 3), this relationship should conform to a slope of unity and y-intercept equal to the latency predicted for each value of the threshold-stimulus angle rate. (D) The coefficient of determination for the unity-line fit for each value of the threshold-stimulus angle rate, (E) the corresponding latency, and (F) sample size (blue). We selected values for latency and the threshold visual-angle rate of change where the coefficient of determination was relatively high (gray bar) for comparison with responses to a live predator (Fig. S2).

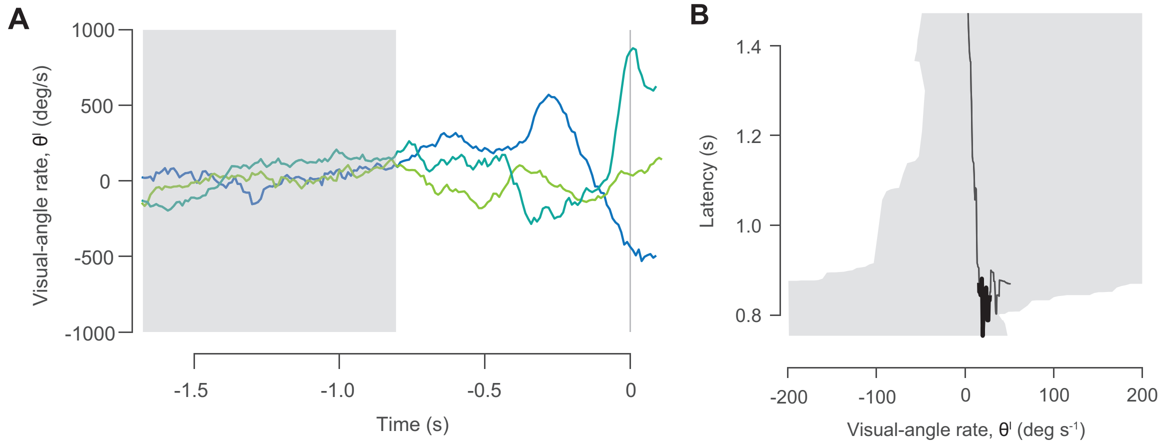

Figure S2: Responses to a live predator. (A–B) Representative measurements of the visual angle and its rate, with time calculated relative to the response time (gray line). We considered all values for the range of latency (gray bars) suggested by measured responses to a projected stimulus (Fig. S1). (C–D) Comparison of measurements from the projected-stimulus experiments (black curves) and live-predator experiments (gray areas) for the threshold-stimulus angle rate for the (C) visual angle and (D) its rate. The margins for the live predator are the first and third quartiles of values for the threshold-stimulus angle rate at each value for the latency. The regions of the threshold-stimulus angle rate with a high coefficient of determination (gray bars in Fig. S1) which fall within the bounds of the live-predator experiments are highlighted (heavy black curves).

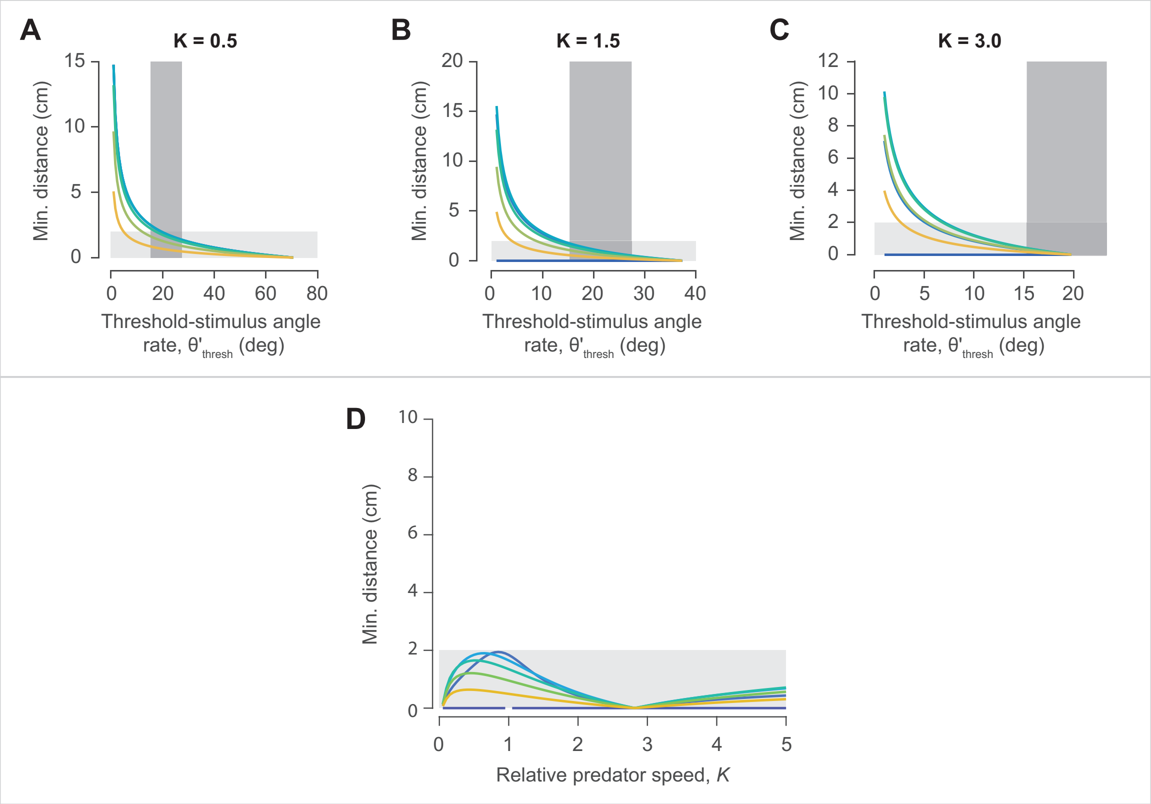

Figure S3: The effects of the threshold-stimulus angle rate on the evasion strategy of zebrafish. (A–C) The minimum distance predicted (Eqn. 5) for varying threshold values for the rate of change of the visual angle. The vertical bars (dark gray) indicate the range of threshold values favored by our analysis of experiments (Fig. S2) the horizontal bars (light gray) indicate distance values where the prey have a low probability of escape ($d_{\mathrm{min}} < 2$ cm). Calculations were performed for predators of variable relative speed ($K = 0.5$, $K = 1.5$, and $K = 3.0$). (D) The minimum distance as a function of relative predator speed at particular values of the threshold visual angle rate ($\theta_{\mathrm{thresh}} = 21$ deg s^{-1}).

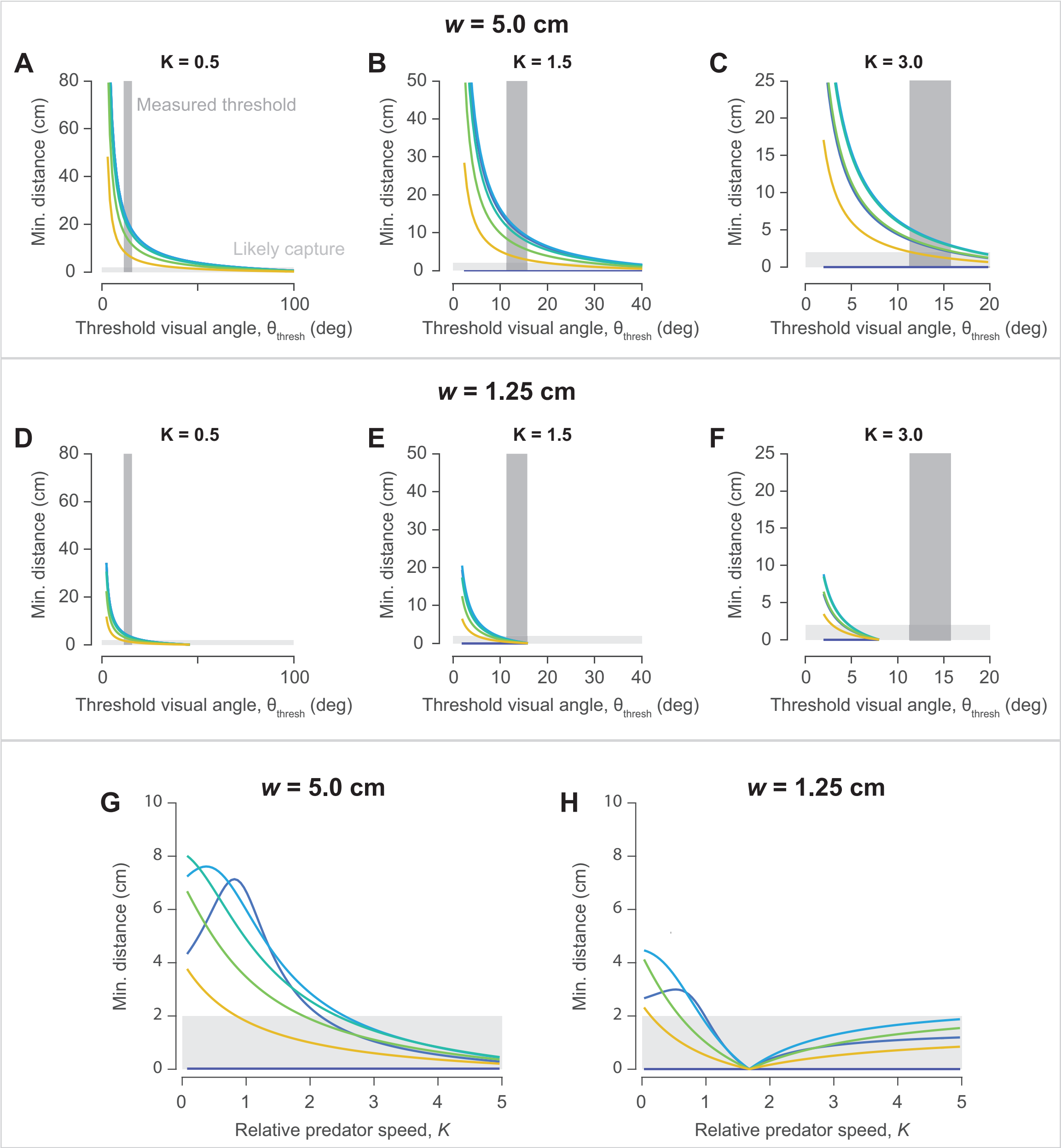

Figure S4: The effects of the threshold-stimulus angle on the evasion strategy of zebrafish for predators of different size. (A–F) The minimum distance predicted (Eqn. 5) for varying threshold values for the rate of change of the visual angle. The vertical bars (dark gray) indicate the range of threshold values favored by our analysis of experiments (Fig. S2) the horizontal bars (light gray) indicate distance values where the prey have a low probability of escape ($d_{\mathrm{min}} < 2$ cm). Calculations were performed for predators of variable relative speed ($K = 0.5$, $K = 1.5$, and $K = 3.0$). Calculations were performed for a relatively wide predator ($w = 5.00$ cm, A–C) and a relatively narrow predator ($w = 1.25$ cm, D–F). (G–H) The minimum distance as a function of relative predator speed at particular values of the threshold-stimulus angle rate ($\theta_{\mathrm{thresh}} = 21$ deg s^{-1}) for a relatively wide predator ($w = 5.00$ cm, G) and a relatively narrow predator ($w = 1.25$ cm, H).
